# Development of Stabilized Growth Factor-Loaded Hyaluronate– Collagen Dressing (HCD) matrix for impaired wound healing

**DOI:** 10.1186/s40824-016-0056-4

**Published:** 2016-04-01

**Authors:** Seong Mi Choi, Hyun Aae Ryu, Kyoung-Mi Lee, Hyun Jung Kim, Ik Kyu Park, Wan Jin Cho, Hang-Cheol Shin, Woo Jin Choi, Jin Woo Lee

**Affiliations:** Brain Korea 21 PLUS Project for Medical Science, Yonsei University, Seoul, South Korea; Department of Orthopaedic Surgery, Yonsei University College of Medicine, Seoul, South Korea; R&D center, Genewel co., Ltd, Sungnam, Korea; School of Systems Biomedical Science, Soongsil University, Seoul, 156-743 Korea

**Keywords:** Stabilized growth factor, HCD matrix, Impaired wound healing

## Abstract

**Background:**

Diabetes mellitus is a disease lack of insulin, which has severely delayed and impaired wound healing capacity. In the previous studies, various types of scaffolds and growth factors were used in impaired wound healing. However, there were several limitations to use them such as short half-life of growth factors *in vivo* and inadequate experimental conditions of wound-dressing material. Thus, our study aimed to determine the biocompatibility and stability of the matrix containing structurally stabilized epidermal growth factor (S-EGF) and basic fibroblast growth factor (S-bFGF).

**Results and Discussion:**

We stabilized EGF and bFGF that are structurally more stable than existing EGF and bFGF. We developed biocompatible matrix using S-EGF, S-bFGF, and hyaluronate– collagen dressing (HCD) matrix. The developed matrix, S-EGF and S-bFGF loaded on HCD matrix, had no cytotoxicity, *in vitro*. Also, these matrixes had longer releasing period that result in enhancement of half-life. Finally, when these matrixes were applied on the wound of diabetic mice, there were no inflammatory responses, *in vivo*. Thus, our results demonstrate that these matrixes are biologically safe and biocompatible as wound-dressing material.

**Conclusions:**

Our stabilized EGF and bFGF was more stable than existing EGF and bFGF and the HCD matrix had the capacity to efficiently deliver growth factors. Thus, the S-EGF and S-bFGF loaded on HCD matrix had improved stability. Therefore, these matrixes may be suitable for impaired wound healing, resulting in application of clinical treatment.

**Electronic supplementary material:**

The online version of this article (doi:10.1186/s40824-016-0056-4) contains supplementary material, which is available to authorized users.

## Background

The wound healing process consists of inflammation, contraction, neoangiogenesis, extracellular matrix deposition, granulation tissue synthesis, re-epithelialization and remodeling. These stages are related in various cellular and molecular signals [1] and when these signals are impaired, the wound healing process is delayed. This phenomenon is caused by the various form of host impairment such as malnutrition, infection and diabetes [2].

Recently, several studies have reported that the growth factors, as therapeutical agents, are widely used in impaired wound healing [3, 4]. Platelet derived growth factor (PDGF), basic fibroblast growth factor (FGF) and epidermal growth factor (EGF) have been most prevalently studied growth factors [5–7]. Indeed, these growth factors are known as stimulating cell proliferation, recruiting various cell types into injured site, and inducing synthesis of extracellular matrix during wound healing process [8–10]. Particularly, the bFGF has ability to promote the wound repair and angiogenesis [11–13] and the EGF contributes to the wound healing via stimulating proliferation and migration of keratinocytes and also facilitating dermal regeneration, *in vivo* [8].

Although these growth factors have therapeutic effects in diabetic wound healing, they have been limited to use as therapeutic agents due to the short half-life of growth factors, *in vivo*. Indeed, the inadequate experimental conditions that did not continuously release growth factors from scaffolds and lose their activity readily when they are loaded onto various types of scaffolds [7, 14–17]. Thus, the new approaches are required for the stable delivery of growth factors locally into the impaired wounds.

There have been variety types of scaffolds such as collagen sponge [18], photo-crosslinking chitosan hydrogel [19] and gelatin sponge [20] which were used to deliver bFGF and EGF onto the diabetic wounds. However, these scaffolds had certain unsatisfied features including rapid absorption and poor mechanical strength [21, 22]. To compensate the shortcomings of the existing wound-dressing materials, the development of new wound-dressing material is necessary.

The hyaluronate- collagen dressing (HCD) matrix has been already used as wound-dressing materials because of advantages such as haemostatic effect, reduction of pain, maintenance of moisturizing condition, and feasible to exchange the wound dressing material because the surface of the HCD matrix does not adhere to wound. For these reason, we considered that HCD matrix is suitable for base material. So, we have assessed the biocompatibility and stability of modified growth factor-loaded HCD matrix, *in vitro*. Furthermore, we investigated *in vivo* stability of modified growth factors-loaded HCD matrix, via application on the diabetic mice.

## Methods

### Materials

Hyaluronic acid (Shisheido, Shizuoka, Japan), Collagen (Koken, Tokyo, Japan) and Pluronic F68 (Daebong LS, Incheon, Korea) are base materials for production of hyaluronate- collagen dressing (HCD) matrix. For the production of HCD matrix, we first dissolved the 0.1 % of collagen in refined water (pH3 ~ 4) and raised pH to 7 ~ 8 then dissolved 0.8 % of HA. The 0.1 % of F68 was added to help blending collagen and HA then evenly mixed them with homogenizer. Subsequently, we added stabilized growth factors with concentrations of 0.1, 0.3, 1, and 2.5ug/cm^2^ and aliquot them into the mold for the lyophilization (Additional file 1: Figure S1). For selection of optimal sterilization method of S-EGF and S-bFGF, we exerted various kinds of methods following ethylene oxide (EO) gas, gamma irradiation (25 kGy) and electronic irradiation. The stabilized epidermal growth factor (S-EGF) and basic fibroblast growth factor (S-bFGF) became more thermostable through structural modification when compared to existing growth factors. The stabilized growth factor loaded HCD matrixes were received from GENEWEL (Seongnam, Gyeonggi-do, Korea).

### MTT Assay and cell proliferation assay

L929 cells (Sigma-Aldrich, St Louis, MO, USA) were cultured in Dulbecco’s Modified Eagle’s Medium-high glucose (DMEM; Gibco, Carlsbad, CA, USA) supplemented with 10 % FBS and 1 % penicillin/streptomycin (P/S). The NIH/3T3 fibroblast cells (Sigma-Aldrich) and Balb/3T3 fibroblast cells (Sigma-Aldrich) were cultured in DMEM (Gibco) supplemented with 0.5 % bovine calf serum (BCS) and 1 % P/S. These cells were incubated at 37 °C incubator with 5 % CO_2_ and medium was replenished every two days. Each of cells were seeded at density of 1X10^5^cells/well on 96 well plates and incubated for 24 h. We classified the groups as negative control (no treatment), positive control (latex glove extracted solution; ISO-10993-5) [23], S-EGF, S-bFGF, and HCD matrix containing S-EGF and S-bFGF and added them in medium and cultured for 48 h. The S-EGF and S-bFGF are loaded on HCD matrix with the concentration of 0.1, 0.3, 1, and 2.5 μg/cm^2^. Then thyiazolyl blue tetrazolium bormide (MTT, M2128-100MG, Sigma-Aldrich) was added and incubated for 3 h. At the indicated time, DMSO was added and absorbance was read at 570 nm.

### Agar overlay

L929 cells were seeded 4X10^5^cells/well on 6well plate. After 24 h of cultivation, medium were removed and 1.5 % agar (BD, Franklin Lakes, NJ, USA) diluted in distilled water was overlaid on the cells. After solidification of agar, 0.01 % neutral red vital dye (Sigma-Aldrich) was treated on medium and placed in dark 37 °C incubator for 90 min. The sterilized paper discs (6 mm) containing HA, were laid on agar and incubated in dark for 24 h. After incubation, cytotoxicity was rated with decolorized zone around the paper discs [24].

### Enzyme-Linked Immunosorbent Assay (ELISA)

Each of disk-shaped HCD matrix containing 1 μg/cm^2^ of S-EGF and S-bFGF (diameter of 10 mm) was immersed in 10 ml of 0.1 % BSA (Sigma-Aldrich) dissolved in PBS and incubated in shaking incubator at 37 °C with 15 rpm for 7 days. Thereafter, to determine the amount of growth factors, these mixtures were diluted to 1/1000 and analyzed by ELISA kit (R&D system, St Cloud, MN, USA). Also, to confirm concentration of released S-EGF and S-bFGF from HCD matrix, we installed matrices (diameter of 30 mm) in the Franz Cell and incubated in shaking incubator at 37 °C with 50 rpm. Then, collected samples on day 1, 3, 7, 10, 14, 21 and confirmed quantity of S-EGF and S-bFGF with ELISA kit (R&D system).

### Preparation of streptozotocin (STZ)-induced type1 diabetic mouse model

ICR mice (Male, aged 7 weeks) were purchased from Orient Bio (Seongnam, Gyeonggi-do, Korea) and housed in wire cage at 20–22 °C at a relative humidity of 40–50 %. We used type1 diabetic mice, induced by intraperitoneal (I.P.) injection of streptozotocin (STZ) (200 mg/kg body weight; Sigma-Aldrich) dissolved in 0.05 M citrate buffer (pH4.5). One week after induction of diabetes, blood glucose levels were measured using OneTouch Select meter (Johnson&Johnson, New Brunswick, NJ, USA). The diabetic phenotype in animals was confirmed by blood glucose levels over 300 mg/dL and maintained for three weeks. The animal experiments were carried out in accordance with guidelines set by the Department of Laboratory Animal Resources, Yonsei University College of Medicine and Seoul, Korea (Permit number = 2015-0190).

### Biocompatibility test of S-EGF and S-bFGF loaded on HCD matrix in type1 diabetic mouse model

STZ-induced type1 diabetic mice (Male, aged 9 weeks) were anesthetized with I.P. injection of Zoletile (30 mg/kg body weight) and Rumpon (10 mg/kg body weight). The hair on the back of mouse was shaved and subsequently wiped with 70 % ethanol. A 10 mm diameter full-thickness of skin wounds were created on the backs of STZ-induced diabetic mice and fixed with a silicone ring to prevent wound contraction. The groups are classified as Defect control, HCD only, HCD+S-EGF 0.3 μg/cm^2^, HCD+S-EGF 1 μg/cm^2^, HCD+S-EGF 2.5 μg/cm^2^, HCD+S-bFGF 0.3 μg/cm^2^, HCD+S-bFGF 1 μg/cm^2^, HCD+S-bFGF 2.5 μg/cm^2^. After application of matrix on wound site, Vaseline gauze (Covidien, St. Louis, MO, USA) and Neo dressing (Everaid, Gangnam, Seoul, Korea) were placed on matrix for the minimal dehydration of wound sites. In each groups, twelve animals were used.

### Statistical analysis

Statistical analysis was performed via Student’s T-TEST and ANOVA. The data was expressed as the mean ± standard deviation (SD). Values of *p < 0.05, **p < 0.01 were considered statistically significant.

## Results and discussion

### Biological stability of S-EGF and S-bFGF

**Fig. 1:**
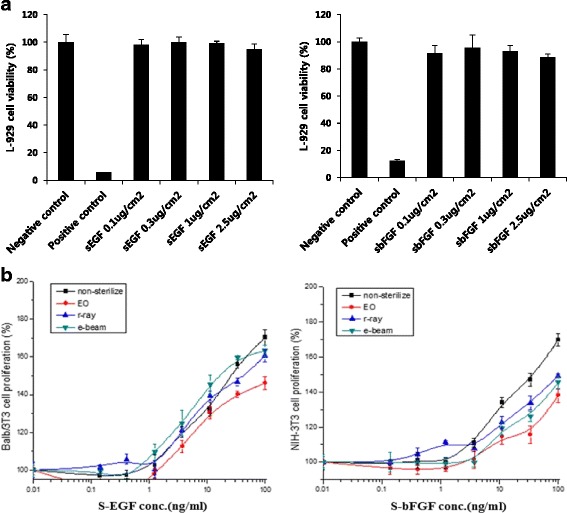
The cytotoxicity stabilized growth factors were tested with various concentrations; 0.1 μg/cm^2^, 0.3 μg/cm^2^, 1 μg/cm^2^ and 2.5 μg/cm^2^ (**a**) and proliferation rate of S-EGF and S-bFGF after sterilization via EO gas, gamma irradiation and electron irradiation (**b**)

First, we performed cytotoxicity assay for the evaluation of the biological stability of stabilized epidermal growth factor (S-EGF) and basic fibroblast growth factor (S-bFGF). All concentrations of S-EGF and S-bFGF have no significance compared to negative control, indicating that S-EGF and S-bFGF do not retain toxicity (Fig. 1a). The result of sterilization, the gamma and electron irradiated S-EGF and S-bFGF showed relatively high proliferation rate compared to EO gas (Fig. 1b). We selected gamma irradiation as our sterilization method because it is better to assure product sterility and also more penetrating than electronic irradiation [25]. The S-EGF and S-bFGF were biologically stable.

### Biological safety of HCD matrix

**Fig. 2:**
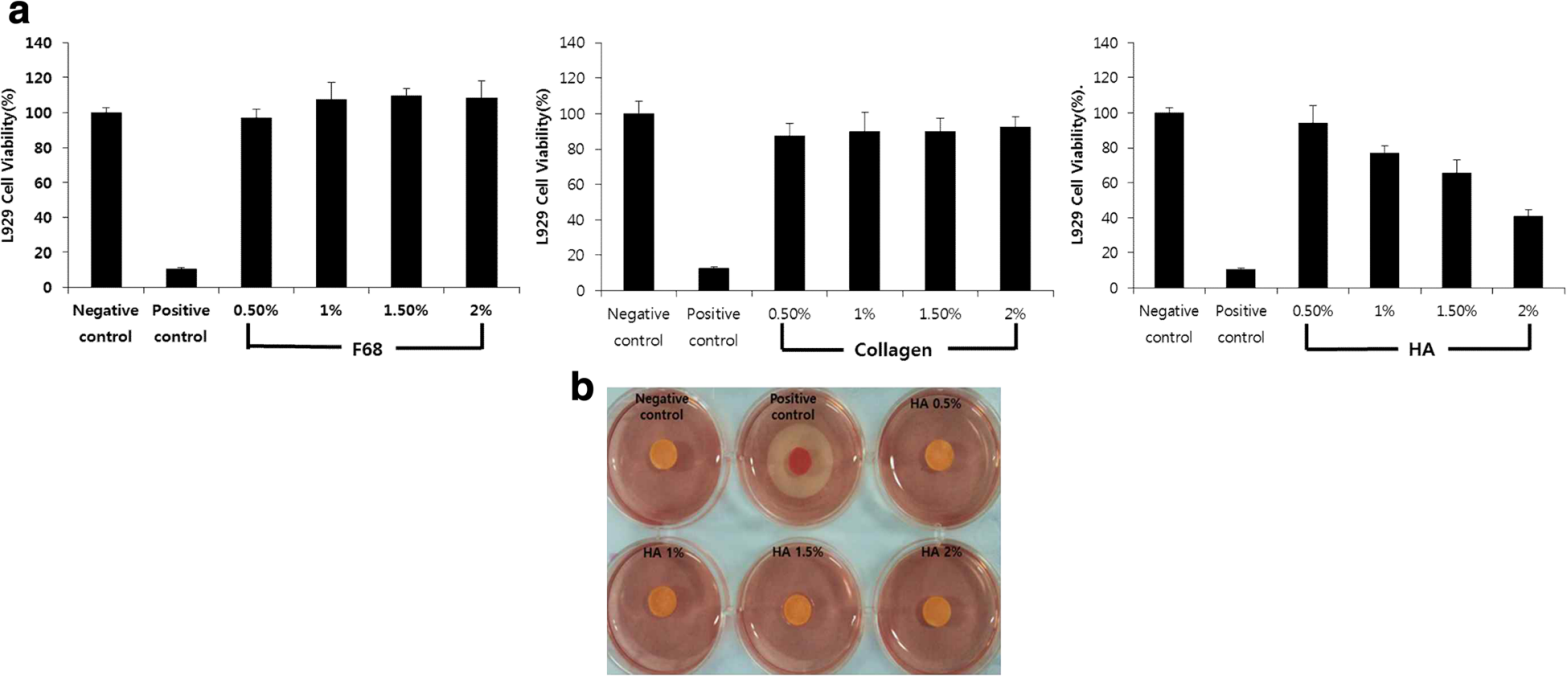
The biocompatibility of base materials of HCD matrix, *in vitro*, was confirmed by cytotoxicity assay of base materials such as F68, collagen and HA (**a**) and the HA was confirmed by agar overlay with various percentages; 0.5 %, 1 %, 1.5 %, and 2 % (**b**)

For biocompatibility of HCD matrix, we evaluated cytotoxicity of three base materials, respectively. The collagen and F68 had more than 90 % of cell survival rate, whereas more than 1 % of HA showed reduction of survival rate (Fig. 2a). Since the cytotoxicity of HA appears to be the characteristics of viscoelasticity rather than cytotoxicity [26, 27], we substituted MTT assay to agar overlay test that is minimally influenced by viscoelasticity. All concentrations of HA showed no decolorized zones, suggesting that HA had no toxicity (Fig. 2b). These data indicate that there is no cytotoxicity in HCD matrix as base material.

### The capacity of S-EGF and S-bFGF loaded on HCD matrix, and its cytotoxicity, *in vitro*

**Fig. 3:**
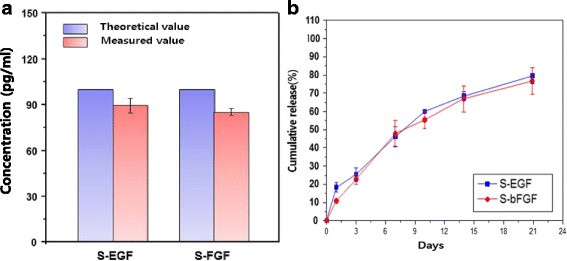
Characteristics of S-EGF and S-bFGF loaded HCD matrix. Amount of S-EGF and S-bFGF contained in HCD matrix was measured via ELISA (**a**) and cumulative release of S-EGF and S-bFGF that are loaded on HCD matrix was measured by ELISA during 21 days (**b**)

We evaluated the final concentration of S-EGF and S-bFGF that are loaded on HCD matrix. As a result, the concentrations were 89.44 ± 4.6 pg/ml and 85.27 ± 2.07 pg/ml, respectively (Fig. 3a). When we evaluated the releasing period of growth factors from HCD matrix, the growth factors were gradually released during long-term period until day21 (Fig. 3b). Thus, this result demonstrated the potential utility of HCD matrix as an agent delivery scaffold.

**Fig. 4:**
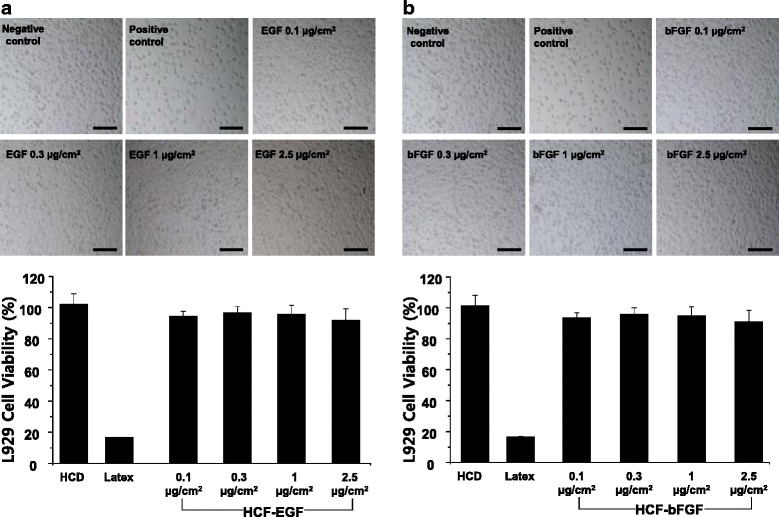
Cytotoxicity of HCD matrix containing S-EGF and S-bFGF was measured with various concentrations of S-EGF (**a**) and S-bFGF (**b**). Scale bar = 200 μm

Additionally, we determined the biological stability of S-EGF and S-bFGF loaded on HCD matrix. Similar to HCD group as a negative control, each concentration of S-EGF and S-bFGF loaded on HCD matrix showed more than 90 % of cell survival rate at all concentrations. These results define the biological safety of our S-EGF (Fig. 4a) and S-bFGF (Fig. 4b) loaded HCD matrix, suggesting the optimal biocompatibility as wound-dressing material.

### The stability of S-EGF and S-bFGF loaded on HCD matrix, *in vivo*

**Fig. 5:**
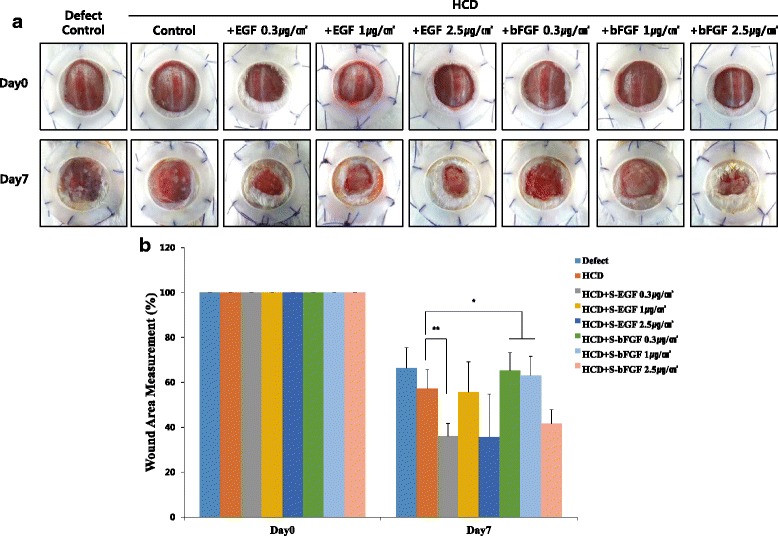
Biological safety of HCD matrix containing S-EGF and S-bFGF, in vivo. The matrix was applied on the wound of STZ-induced diabetic mice with following groups; defect control, HCD control, HCD + S-EGF 0.3 μg/cm^2^, HCD + S-EGF 1 μg/cm^2^, HCD + S-EGF 2.5 μg/cm^2^, HCD + S-bFGF 0.3 μg/cm^2^, HCD + S-bFGF 1 μg/cm^2^, and HCD + S-bFGF 2.5 μg/cm^2^. The macroscopic representative images were taken on day 0 and 7 (**a**) and quantitative analysis of wound area is measured (**b**). *p < 0.05, **p < 0.01

Next, we assessed the stability of S-EGF and S-bFGF loaded on HCD matrix *in vivo*. These matrixes had no inflammatory effect at day 7, indicating that the S-EGF and S-bFGF loaded on HCD matrix is negligent as wound-dressing material. In addition, the S-EGF and S-bFGF loaded on HCD matrix showed slight acceleration of wound healing when compared to defect control and HCD matrix only group (Fig. 5a). As shown in Fig. 5b, the wound areas of S-EGF group were decreased about 50 % compared with defect control group (Fig. 5b). Thus, these results suggest that S-EGF and S-bFGF loaded on HCD matrix is biologically safe to use as wound-dressing material and have the potential in acceleration of wound healing.

Taken together, our results demonstrated that S-EGF and S-bFGF are more stabilized than existing EGF and bFGF after modification, resulting in longer half-life and releasing period of growth factors from HCD matrix, *in vitro*. Indeed, this matrix was biologically safe, *in vivo*. Although, the further studies toward S-EGF and S-bFGF loaded on HCD matrix are needed to evaluate the effectiveness, *in vivo*, S-EGF and S-bFGF loaded on HCD matrix is expected to be valuable wound-dressing material in impaired wound healing.

## Conclusions

For impaired wound healing, we developed the S-EGF and S-bFGF loaded on HCD matrix, which has no effects on cytotoxicity and proliferation. Also, we confirmed stability and releasing period of S-EGF and S-bFGF loaded on HCD matrix. As a result, these matrixes showed more stabilized and longer releasing period of growth factors than existing EGF and bFGF, *in vitro*. Furthermore, when these matrixes were applied on the wound of diabetic mice, the inflammatory response was not occurred, indicating that this matrix is biocompatible. According to these results, the S-EGF and S-bFGF loaded on HCD matrix may overcome the disadvantages of established scaffolds in wound healing study. Taken together, the S-EGF and S-bFGF loaded on HCD matrix may contribute to the application in impaired wound healing because of the biological safety.

## Additional file

Additional file 1: Figure S1. The fabrication process of S-EGF and S-bFGF loaded HCD matrix with collagen, HA, and F68. (PDF 42 kb)

## Abbreviations

F68: pluronic 68
HA: hyaluronic acid
HCD: Hyaluronate- Collagen Dressing
S-bFGF: stabilized-basic fibroblast growth factor
S-EGF: stabilized-epidermal growth factor
