# Supplementary figures and images for: Development of Stabilized Growth Factor-Loaded Hyaluronate– Collagen Dressing (HCD) matrix for impaired wound healing
Source: Biomater Res. 2016 Apr 1;20:9. doi: 10.1186/s40824-016-0056-4 (PMC4818407; doi:10.1186/s40824-016-0056-4)

Supplementary figure 1

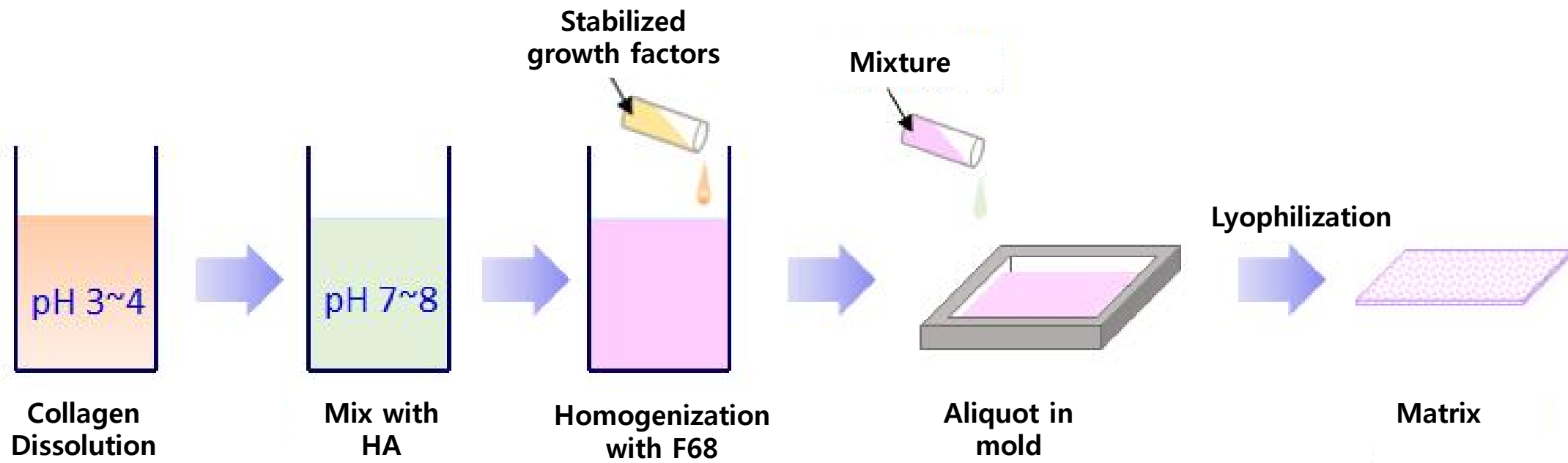

Supplement: Additional file 1: Figure S1. — The fabrication process of S-EGF and S-bFGF loaded HCD matrix with collagen, HA, and F68. (PDF 42 kb) [file 40824_2016_56_MOESM1_ESM.pdf]
